# Supplementary material for: The GPR4 antagonist NE-52-QQ57 increases survival, mitigates the hyperinflammatory response and reduces viral load in SARS-CoV-2-infected K18-hACE2 transgenic mice
Source: Front Pharmacol. 2025 Jul 9;16:1549296. doi: 10.3389/fphar.2025.1549296 (PMC12283652; doi:10.3389/fphar.2025.1549296)

Supplementary Figure 1

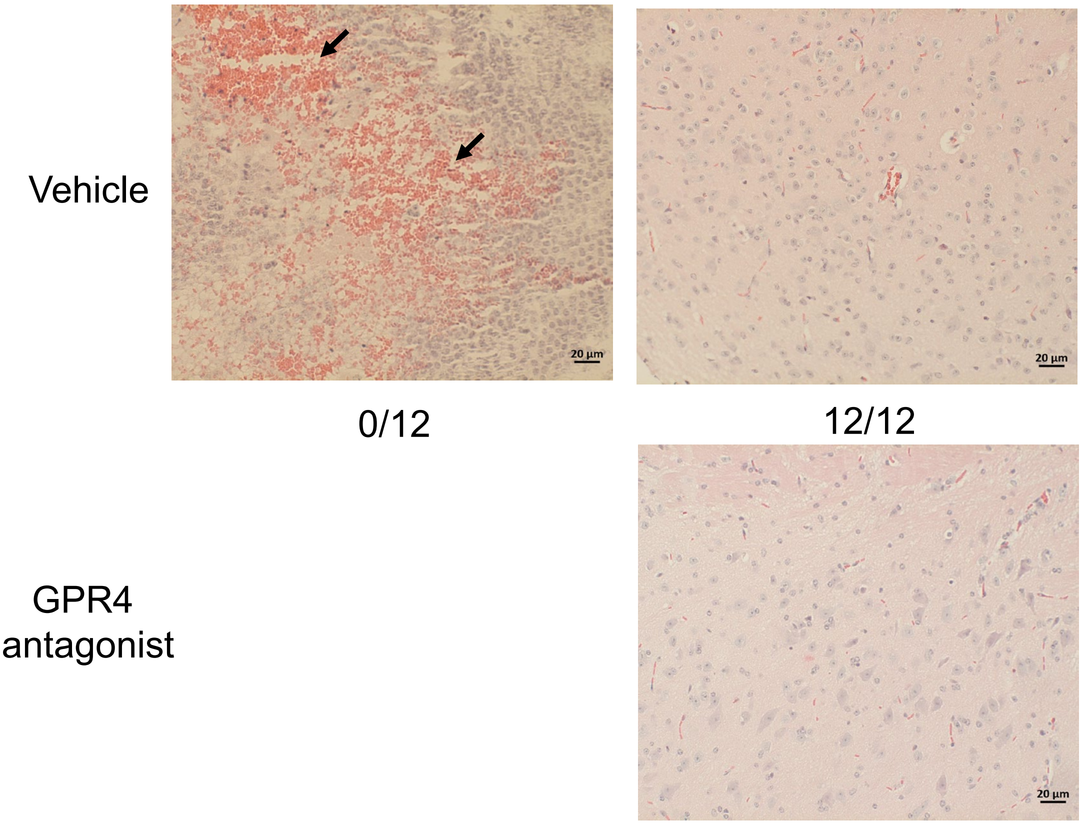

Supplementary Figure 2

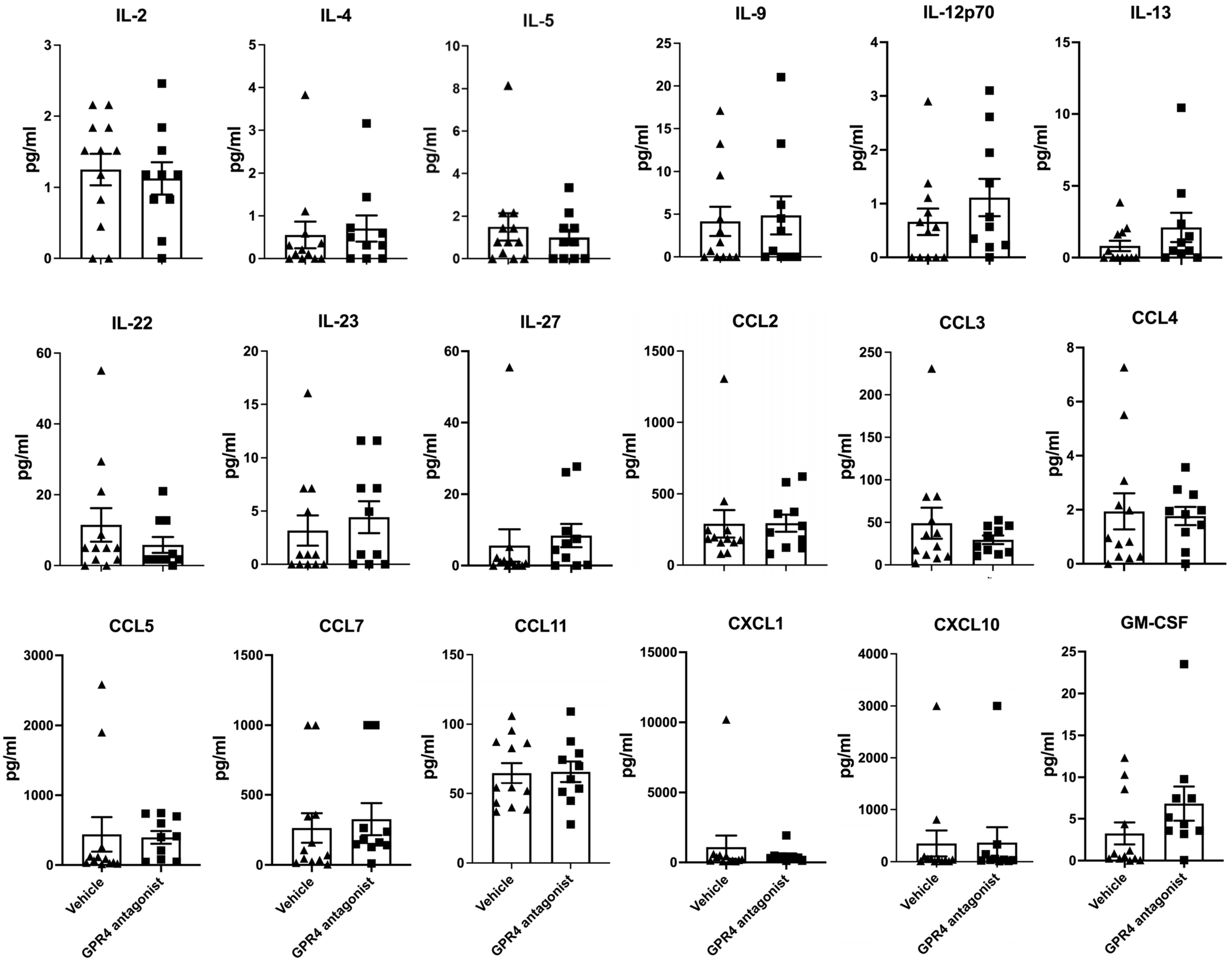

Supplementary Figure 3

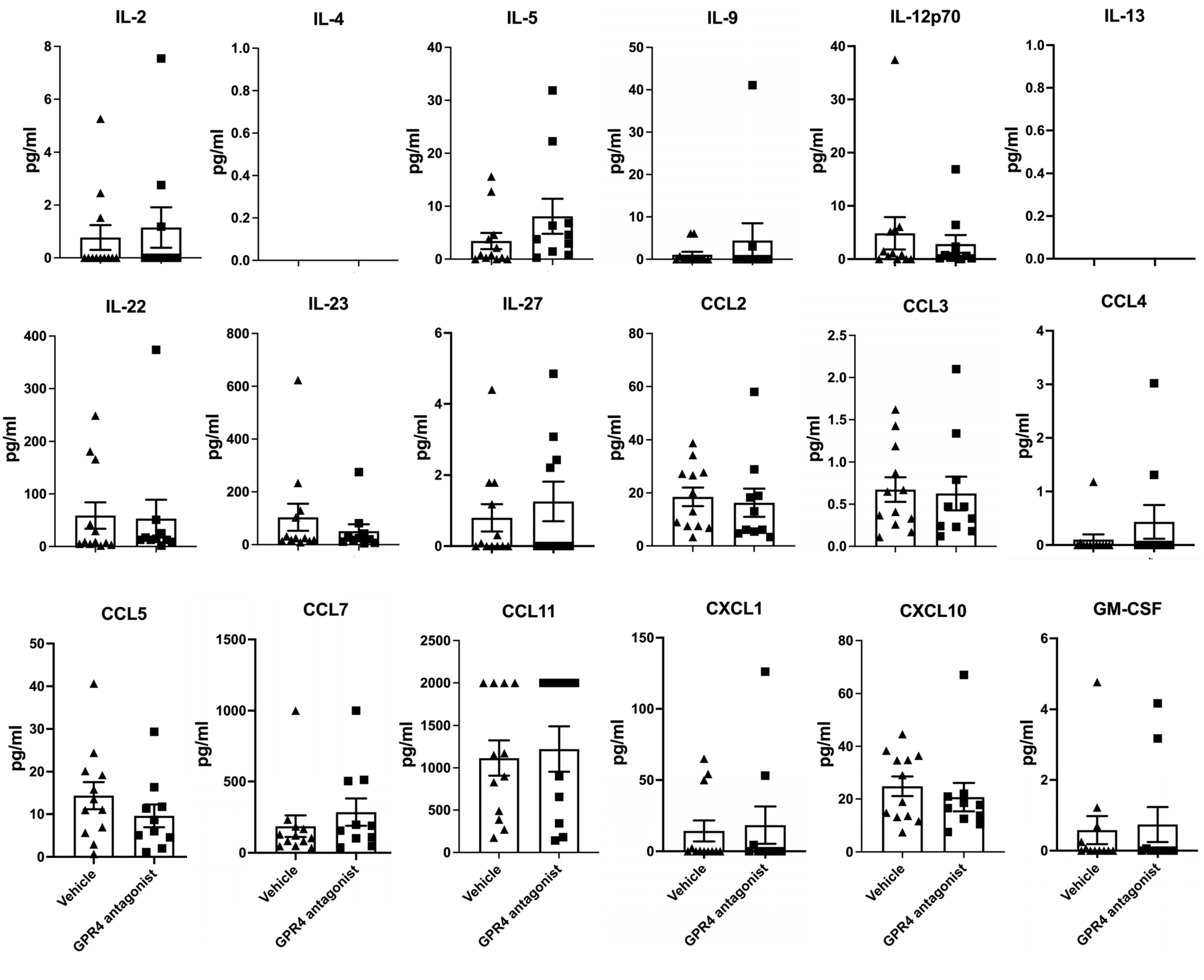

Supplementary Figure 4

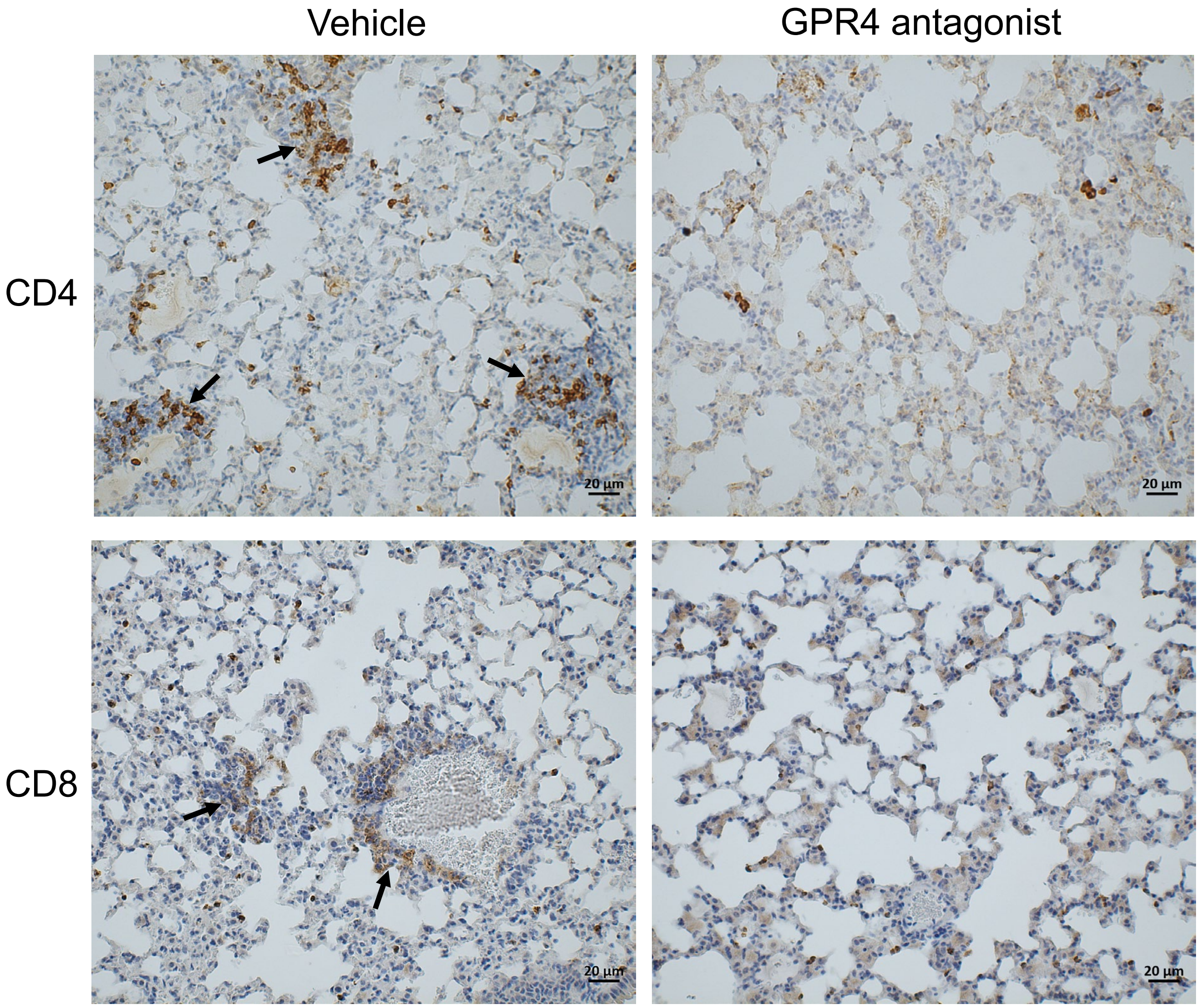

Supplementary Figure 5

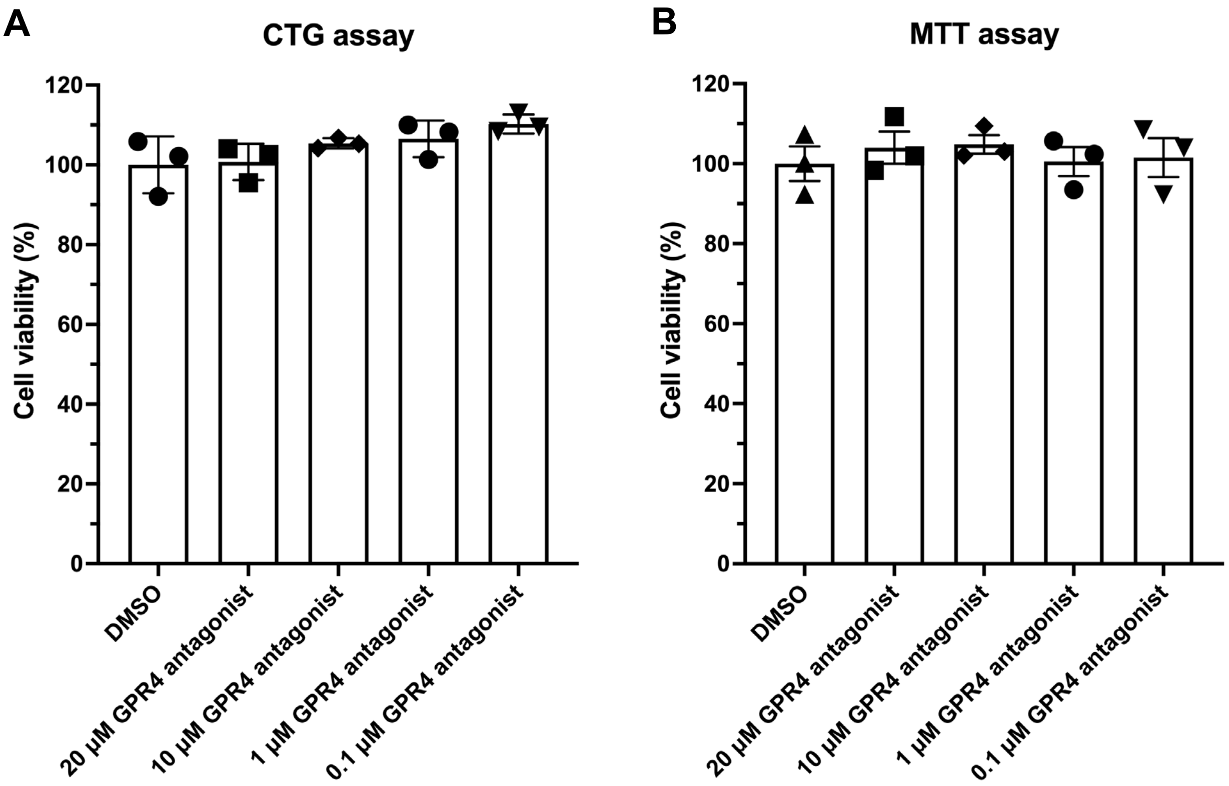

Supplementary Figure 6

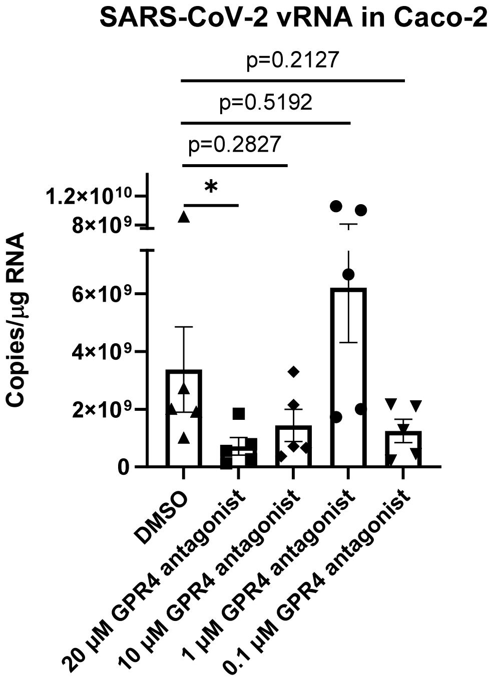

Suppl. Fig. 7

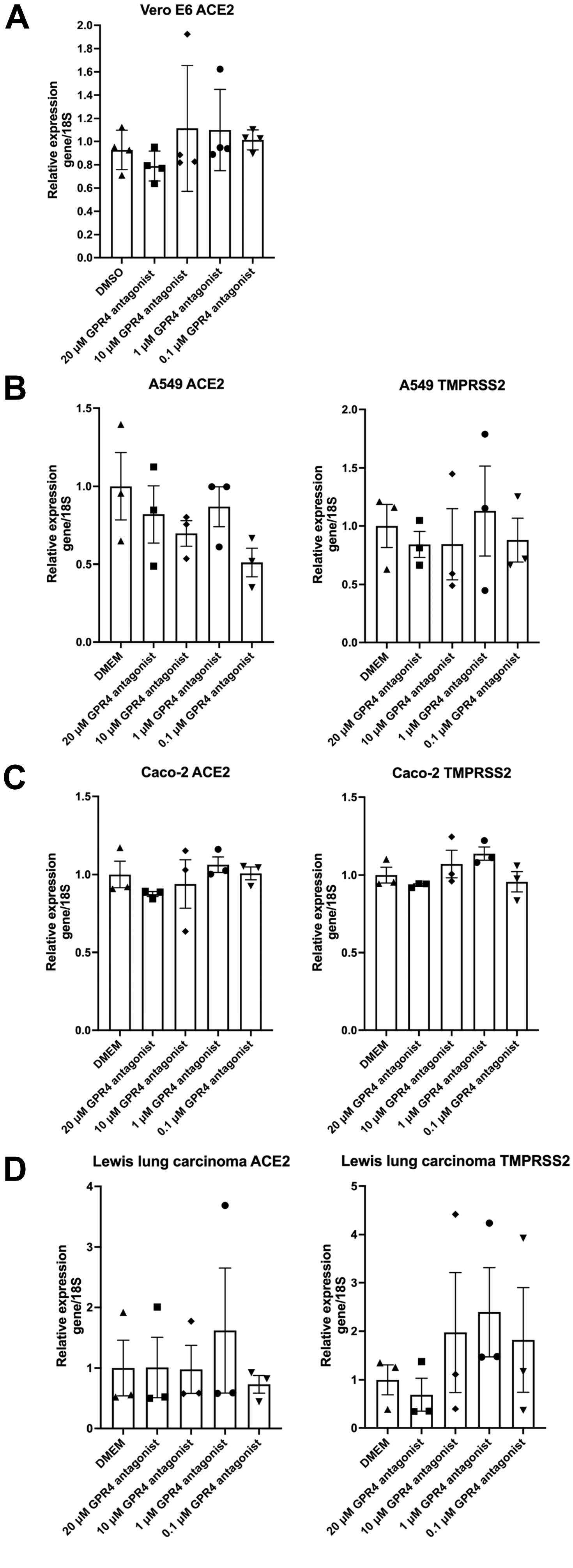
A
Vero E6 ACE2
Relative expression gene/18S
DMSO
20 µM GPR4 antagonist
10 µM GPR4 antagonist
1 µM GPR4 antagonist
0.1 µM GPR4 antagonist
B
A549 ACE2
A549 TMPRSS2
DMEM
C
Caco-2 ACE2
Caco-2 TMPRSS2
D
Lewis lung carcinoma ACE2
Lewis lung carcinoma TMPRSS2

Supplementary Figure 8

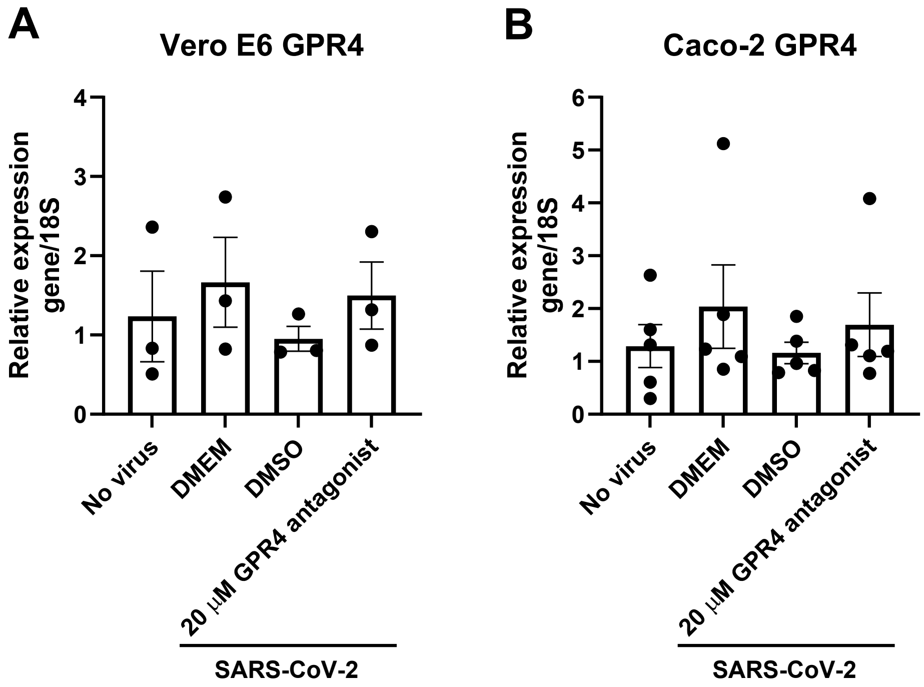

Supplement: Supplementary file 1 [file DataSheet1.pdf]
